# Identification of CD8^+ ^cytotoxic T lymphocyte epitopes from porcine reproductive and respiratory syndrome virus matrix protein in BALB/c mice

**DOI:** 10.1186/1743-422X-8-263

**Published:** 2011-05-30

**Authors:** Weijun Zhang, Yan Lin, Yu Bai, Tiegang Tong, Qun Wang, Nihong Liu, Guangliang Liu, Yihong Xiao, Tao Yang, Zhigao Bu, Guangzhi Tong, Donglai Wu

**Affiliations:** 1 The Key Laboratory of Veterinary Public Health, Ministry of Agriculture, State Key Laboratory of Veterinary Biotechnology, Harbin Veterinary Research Institute, Chinese Academy of Agricultural Sciences, Harbin 150001, PR China; 2 Shanghai Veterinary Research Institute, Chinese Academy of Agricultural Sciences, Shanghai 200241, PR China; 3 Graduate School of Chinese Academy of Agricultural Sciences, Beijing 100081, PR China

**Keywords:** Porcine reproductive and respiratory syndrome virus, Matrix protein, CTL epitopes, Intracellular cytokine staining, ELISPOT

## Abstract

Twenty-seven nanopeptides derived from the matrix (M) protein of porcine reproductive and respiratory syndrome virus (PRRSV) were screened for their ability to elicit a recall interferon-γ (IFN-γ) response from the splenocytes of BALB/c mice following DNA vaccination and a booster vaccination with recombinant vaccinia virus rWR-PRRSV-M. We identified two peptides (amino acid residues K_93_FITSRCRL and F_57_GYMTFVHF) as CD8^+ ^cytotoxic T lymphocyte (CTL) epitopes. These peptides elicited significant numbers of IFN-γ secreting cells, compared with other M nonapeptides and one irrelevant nonapeptide. Bioinformatics analysis showed that the former is an H-2K*^d^*-restricted CTL epitope, and the latter is an H-2D*^d^*-restricted CTL epitope. Multiple amino acid sequence alignment among different PRRSV M sequences submitted to GenBank indicated that these two CTL epitopes are strongly conserved, and they should therefore be considered for further research on the mechanisms of cellular immune responses to PRRSV.

## 1. Introduction

Porcine reproductive and respiratory syndrome virus (PRRSV) is one of the most important swine viral pathogens, and has caused significant economic losses to the swine industry worldwide. Characterization of field isolates suggested that PRRSV are genetically diverse, and this genetic variation increases the difficulty of developing effective vaccines. Based on significant sequence differencesPRRS viruses are grouped into two distinct genotypes, European isolate (Lelystad virus, LV) and North American isolate (VR-2332) [1]PRRSV has two major structural proteins, GP5 and M, encoded by ORFs 5 and 6, respectively. GP5, the most important neutralizing antigen of PRRSV, has the highest genetic diversity among isolates [2]. And, recent studies in Yorkshire × Landrace crossed and outbred pigs, showed that there are two immuno-dominant T-cell epitopes derived from the GP5 protein: L_117_AALICFVIRLAKNC and K_149_GRLYRWRSPVII/VEK [3]. The M protein, which contains highly conserved amino acid sequences, also has very good immunogenicity and is associated with protection against PRRSV infection. DNA vaccinations have also revealed that M is the most potent inducer of T lymphocyte proliferation [4].

At present, effective vaccination strategies for the prevention and control of PRRSV infection are not available. Vaccines based on inactivated PRRSV virus have been ineffective at inducing protective immune responses. Live-attenuated PRRSV vaccines can provide protection against this pathogen, but have been observed to revert to virulence [5], restricting the application of this vaccination approach. The rational development of future PRRSV vaccines will necessitate a systematic understanding of the protective humoral and cellular immune responses that occur during PRRSV infection, and should aim to induce a broad immune responses that accommodates the plasticity of the major antigenic sites. Recent research has indicated that cell-mediated immunity may play a very important role in the clearance of PRRSV [6]. Major histocompatibility complex (MHC) I restricted cytotoxic T lymphocytes (CTLs) can kill virus-infected cells and eliminate potential sources of new virus [7]. Hence, identification of CTL epitopes is crucial in the design of synthetic vaccines, and a number of studies have successfully identified pathogen-derived T cell epitopes [8-11].

Unlike many other pathogens, there is limited knowledge of the specific PRRSV-derived peptides targeted by T-cells. In the current study, we report the identification of CTL epitopes from the PRRSV (Ch-1a strain) M protein in a mouse model. We screened peptides derived from the PRRSV M protein for their ability to induce interferon (IFN)-γ in splenocytes harvested from BALB/c mice following DNA vaccination and a booster vaccination with recombinant vaccinia virus expressing M protein. The screen identified two peptides that elicited IFN-γ production in CD3^+^CD8^+ ^splenocytes of vaccinated mice. A multiple amino acid sequence alignment among different PRRSV M proteins indicates that these two peptides are strongly conserved across multiple PRRSV strains and therefore should be considered for further research.

## 2. Materials and methods

### 2.1. Viruses and cell lines

The PRRSV Ch-1a strain, the vaccinia virus WR strain, and the Akabane virus OBE-1 strain were part of our laboratory collection. The former was propagated in Marc-145 cells, and the latter two were propagated in BHK-21 cells, respectively, and these two cell lines were cultured in DMEM (Invitrogen) supplemented with 10% of fetal calf serum (FCS, Invitrogen) in a humidified 37°C, 5% CO_2 _incubator.

### 2.2. Cloning of PRRSV M and murine ubiquitin (Ub) genes

Total RNA was extracted from the PRRSV CH-1a strain and the spleens of BALB/c mice. Full length cDNAs were amplified based on the complete open reading frames (ORFs) of M and ubiquitin (Ub) following reverse transcription (GenBank accession numbers AY032626 and AZ033428) using the M-F and M-R primers pair or the Ub-F and Ub-R primer pair (Table 1). The full length cDNAs were cloned into the pMD18-T vector (TaKaRa) and are referred to as pMD-M and pMD-U, respectively.

**Table 1:** Primers designed for PCR

primer	Sequence (5'-3')	Note
M-F	CCC*AAGCTT*AGC**ATG**GGGTCGTCTCTAGA	*Hind *III
M-R	CCG*CTCGAG***CTA**TTTGGCATATTTGACA	*Xho*I
Ub-F	TCA*GGATCC*GCCGCCGCC**ATG**CAGATTTTCGTG	*BamH *I
Ub-R	GGAGCCTGGAGAAGCACCTCTCAGGCGAAGGAC	
Truncated-M-F	CCG*GAATTC*ATGGGAGTGTACTCGGCCATAG	*EcoR *I
Truncated-M-R	CCG*CTCGAG*CTATTTGGCATATTTGAC	*Xho *I
Fusion-M-Ub-F	TCTCCAGGCTCCATGGGGTCGTCTCTA	*Linker*
Fusion-M-Ub-R	GGAGCCTGGAGAAGCACCTCTCAGGCGAAGGAC	*Linker*
pSC11-M-F	ATA*GTCGAC*GCCGCCGCC**ATG**GGGTCGTCTCTAG	*Sal*I
pSC11-M-R	GCC*GTCGAC***CTA**TTTGGCATATTTGACAAGGTTTAC	*Sal*I
P7.5-F	GCACGGTAAGGAAGTAGAAT	

Note: The bold letter of primers are start or stop codons; the underlined sequence of primers represents the Kozak motif or the linker sequence.

### 2.3. The expression of a truncated PRRSV M gene

An indirect enzyme-linked immunosorbent assay (iELISA) method was established to evaluate specific antibody against M protein. A truncated M protein (85-174aa), which was used as the coating antigen, was expressed and purified using a prokaryotic expression system. The truncated M gene cDNA was amplified from pMD-M using the truncated-M-F and truncated-M-R primers (Table 1), ligated into pGEX-6P-1 (GE Healthcare), and subsequently named pGEX-M. For protein expression, pGEX-M was transformed into *E.coli *BL21 (DE3) and recombinant protein expression was induced with 0.5 mM IPTG. The samples were harvested by centrifugation and the pellets were resuspended with phosphate buffered saline (PBS, PH 8.0). After being analyzed by sodium dodecyl sulfatepolyacrylamide gel electrophoresis (SDS-PAGE) and Western blot with anti-PRRSV-M monoclonal antibody (our laboratory collection), the fusion-expressed truncated M protein was purified by GST tag according to the manufacturer's instructions (GE Healthcare). Finally, the concentration of the purified protein was determined using a Bradford kit (Bio-Rad) according to the manufacturer's instructions.

### 2.4. Construction of the eukaryotic expression plasmid pVAX1-Ub-M

The Ub and M genes were fused using splicing by overlapping extension PCR (SOE-PCR). The Ub and M genes were amplified from pMD-U and pMD-M with the primer pairs Ub-F and Fusion-M-Ub-R and Fusion-M-Ub-F and M-R, respectively (Table 1). The fusion gene product, referred to as Ub-M, was amplified from the purified PCR products with Ub-F and M-R and inserted into the eukaryocyte expression vector pVAX1, and was named pVAX1-Ub-M.

### 2.5. Expression of pVAX1-Ub-M in vitro

*In vitro *expression of the Ub-M was demonstrated by transient transfection of BHK-21 cells using FuGene 6 (Roche) according to the manufacturer's instructions. Ub-M protein expression was detected by indirect immunofluorescence analysis (IFA). An anti-PRRSV-M monoclonal antibody and a FITC-conjugated rabbit anti-mouse IgG secondary antibody (Sigma) were used for IFA detection of M protein. Fluorescence was observed using a fluorescence microscope (LEICA DM IRE2).

### 2.6. Construction of recombinant vaccinia virus

#### 2.6.1. Construction of the transferring plasmid pSC11-M

The transferring vector pSC11 (our laboratory collection), which is composed of the early promoter P7.5 and late promoter P11 of vaccinia virus, and the LacZ and Amp genes controlled by the promoter P11 as the reporter genes, was used in this study. To construct the transferring vector pSC11-M, the complete M gene was amplified with the pSC11-M-F and pSC11-M-R primer pair (Table 1) and inserted into the pSC11. The primer P7.5-F (Table 1), derived from promoter P7.5 sequence, was used for directional identification and the positive clones, which were subsequently named pSC11-M.

#### 2.6.2. Homologous recombination and selection of the recombinant virus

Homologous recombination was performed by lipofectin-mediated co-transfection of the transferring plasmid pSC11-M and the WR strain vaccinia virus into 80% confluent TK^-^143 cells cultured in MEM medium containing 25 μg/ml BrdU (Sigma). The viruses were collected after the appearance of cytopathic effect (CPE), and recombinant vaccinia virus was purified according to the expression of LacZ gene.

### 2.7. Identification of the recombinant virus

#### 2.7.1. Western blot analysis of the recombinant virus

Western blot analysis was performed as described previously [12]. Briefly, a BHK-21 cell layer was infected with rWR-PRRSV-M recombinant vaccinia virus or the vaccinia virus WR strain at a multiplicity of infection (MOI) of 0.1. Cells were harvested 3 days post-infection, and total cell lysates were prepared with lysis buffer (10 mM Tris-Cl pH 7.4, 1 mM MgCl_2_, 0.5% NP40, 20 μg/ml DNase I). Cell lysates were separated by SDS-PAGE and were subsequently transferred to a membrane for Western blot analysis using an anti-PRRSV-M monoclonal antibody, HRP-conjugated goat anti-mouse IgG secondary antibody (Bio-Rad), and DAB substrate. The positive recombinant virus was named rWR-PRRSV-M.

#### 2.7.2. IFA of the recombinant virus

The expression of M was further confirmed by IFA. Briefly, BHK-21 cells were infected with either rWR-PRRSV-M or the vaccinia virus WR strain when the BHK-21 cells reached 70-80% confluence in a 24-well plate. M protein expression was evaluated by IFA 3 days post infection using the anti-PRRSV-M monoclonal antibody followed by a FITC-conjugated rabbit anti-mouse IgG secondary antibody (Sigma). Specific fluorescence was observed using a fluorescence microscope (LEICA DM IRE2).

### 2.8. Prediction and synthesis of M protein CTL epitopes in BALB/c mice

The sequences of M were screened for potential H2-K*^d^*/H2-D*^d^*/H2-L*^d ^*9-mer epitopes using the algorithms from the SYFPEITHI website [13], the HLA peptide binding prediction website (BIMAS) [14] and PRED^BALB/c ^[15]. The 27 peptides (Table 2), with highest binding score (BS) as predicted by each algorithm, were synthesized by Scilight Biotechnology LLC (Beijing, China) and purified to a purity > 95% using high performance liquid chromatography (HPLC).

**Table 2:** Predicted M protein peptides chemically synthesized and screened to identify CTL epitopes

Bioinformatics Methods						
	**SYFPEITHI**		**BIMAS**		**PRED**^**BALB/c**^	

	**Peptides**	**Bingding score**	**Peptides**	**Bingding score**	**Peptides**	**Bingding score**

H-2K*^d^*	K_93_FITSRCRL	BS = 23	K_93_FITSRCRL	BS = 2304.000	K_93_FITSRCRL	BS = 9.70
	T_25_YTPVMIYA	BS = 24	T_25_YTPVMIYA	BS = 72.000	T_25_YTPVMIYA	BS = 5.3
	S_12_TAPQKVLL	BS = 20	S_12_TAPQKVLL	BS = 57.600	S_12_TAPQKVLL	BS = 6.10
	S_140_TTVNGTLV	BS = 19	S _140_TTVNGTLV	BS = 14.400	S _140_TTVNGTLV	BS = 6.10
	T_146_LVPGLKGL	BS = 19	T_146_LVPGLKGL	BS = 115.200	T_146_LVPGLKGL	BS = 9.10
	A_21_FSITYTPV	BS = 18	A_21_FSITYTPV	BS = 345.600	A_21_FSITYTPV	BS = 9.54
	R_40_LLGLLHLL	BS = 18	R_40_LLGLLHLL	BS = 96.00	R_40_LLGLLHLL	BS = 7.80
	L_34_KVSRGRLL	BS = 17	L_34_KVSRGRLL	BS = 9.600	L_34_KVSRGRLL	BS = 5.64
	H_64_FQSTNRVA	BS = 17	H_64_FQSTNRVA	BS = 28.800	H_64_FQSTNRVA	BS = 5.64
	C_102_LLGRKYIL	BS = 17	C_102_LLGRKYIL	BS = 96.00	C_102_LLGRKYIL	BS = 7.84
	Q_16_KVLLAFSI	BS = 16	L_162_KVSRGRLL	BS = 11.520	L_162_KVSRGRLL	BS = 5.80
	L_162_KVSRGRLL	BS = 16	L_162_KVSRGRLL	BS = 0.012	L_162_KVSRGRLL	BS = 6.10
	S_23_ITYTPVMI	BS = 14	S_23_ITYTPVMI	BS = 48.000	S_23_ITYTPVMI	BS = 8.50

H-2D*^d^*			Y_26_TPVMIYAL	BS = 20.000	Y_26_TPVMIYAL	BS = 9.82
			F_57_GYMTFVHF	BS = 7.200	F_57_GYMTFVHF	BS = 10.00
			T_13_APQKVLLA	BS = 12.000	T_13_APQKVLLA	BS = 8.58
			Q_164_GVVNLVKY	BS = 2.000	Q_164_GVVNLVKY	BS = 9.40
			F_122_HPIAANDN	BS = 2.400	F_122_HPIAANDN	BS = 9.20
			P_138_GSTTVNGT	BS = 0.200	P_138_GSTTVNGT	BS = 9.20

H-2L*^d^*	A_14_PQKVLLAF	BS = 24	A_14_PQKVLLAF	BS = 450.000	A_14_PQKVLLAF	BS = 9.38
	V_148_PGLKGLVL	BS = 24	V_148_PGLKGLVL	BS = 150.000	V_148_PGLKGLVL	BS = 9.50
	D_11_STAPQKVL	BS = 20	D_11_STAPQKVL	BS = 25.000	D_11_STAPQKVL	BS = 7.50
	Y_86_SAIETWKF	BS = 20	Y_86_SAIETWKF	BS = 50.000	Y_86_SAIETWKF	BS = 7.50
	E_117_SAAGFHPI	BS = 20	E_117_SAAGFHPI	BS = 6.500	E_117_SAAGFHPI	BS = 4.70
	T_96_SRCRLCLL	BS = 19	T_96_SRCRLCLL	BS = 7.500	T_96_SRCRLCLL	BS = 7.60
	G_139_STTVNGTL	BS = 18	G_139_STTVNGTL	BS = 25.000	G_139_STTVNGTL	BS = 7.40
	F_22_SITYTPVM	BS = 15	F_22_SITYTPVM	BS = 25.000	F_22_SITYTPVM	BS = 7.00

Note: The 27 peptides with highest binding score (BS) as predicted by each algorithm are shown, starting with the peptide giving the highest score at the top for each protein. Sequences of 9 mers are given from BIMAS, SYFPEITHI and PRED^BALB/c ^predictions, respectively. The N-terminal amino acid position is indicated for epitopes predicted from the whole M protein.

### 2.10. Vaccination using a DNA prime-recombinant vaccinia virus boost strategy

All animal experiments were performed according to national and institutional guidelines. One hundred and ninety female BALB/c mice (Vital River Laboratory Animal Technology Co., Beijing, China) were maintained in isolation cages at the Experimental Animal Center of Harbin Veterinary Research Institute (Harbin, China). Mice were divided into three groups: the pVAX1-Ub-M vaccination group (n = 120), the pVAX1 control vaccination group (n = 35) and the PBS control vaccinated group (n = 35).

The plasmid DNA used for immunization was purified using the EndoFree Mega plasmid preparation kit (Qiagen). The pVAX1-Ub-M and pVAX1 cohorts were intramuscularly (i.m.) vaccinated with 100 μg pVAX1-Ub-M or pVAX1 plasmid DNA in 100 μl PBS, respectively. The PBS control group received an i.m. injection of 100 μL PBS. Each group was vaccinated four times at 3-week intervals. To enhance the specific CTL responses to M protein, the mice received an intraperitoneal (i.p.) injection containing 0.1 MOI of rWR-PRRSV-M on day 7 following the fourth DNA vaccination. At the same time, five mice from the pVAX1 and PBS vaccination groups were also inoculated intraperitoneally with 0.1 MOI of rWR-PRRSV-M on day 7 following the fourth vaccination in order to compare the specific antibody raised against M protein of different experimental groups following vaccination with rWR-PRRSV-M. All procedures were conducted with the protocols approved by Experimental Animal Center of Harbin Veterinary Research Institute (HVRI) of the Chinese Academy of Agricultural Sciences (CAAS).

### 2.11. ELISA antibody response in mice after immunization

To investigate the specific antibody response, serum samples was obtained from vaccinated mice 7 days after each DNA vaccination and 3 days after boosting with rWR-PRRSV-M. An iELISA was performed according to methods described previously [16]. The purified M protein was used as the coating antigen, the tested sera applied at a 1:10 dilution, and an HRP-conjugated goat anti-mouse IgG antibody (Bio-Rad) used as the secondary antibody. The microplates were developed using ortho-phenylene diamine (OPDA, AMERSCO) and H_2_O_2 _for 10 min, after which the reaction was stopped by the addition of 1 M H_2_SO_4_. Finally, the optical density (OD) was read at 492 nm. An anti-PRRSV-M monoclonal antibody was used as a positive control. Serum containing antibodies against Akabane virus and the sera of control group mice served as negative controls.

### 2.12. In vitro stimulation of splenocytes

Isolated splenocytes were added to U-bottomed 96-well plates (Corning Inc) at 10^6 ^cells/well in 100 μL complete RPMI-1640 medium supplemented with 10% FCS. The cells were then mixed with 100 μl media containing a nonamer peptide derived from PRSSV M protein at 20 μg/ml, or phorbol-12-myristate-13-acetate (PMA 10 ng/mL) and ionomycin (500 ng/mL) as a positive control. Cells incubated in medium alone or with a peptide derived from the infectious bronchitis virus (IBV) H52 strain [17] were used as negative controls. Following a 12 h incubation at 37°C, 10 uM monensin (Sigma) was added and the splenocytes were incubated for an additional 4 h at 37°C before staining.

### 2.13. Surface antigen staining and intracellular cytokine staining (ICS)

The number of CD3^+^CD8^+ ^T cells producing IFN-γ on days 3, 5 and 12 after boosting with rWR-PRRSV-M was determined using flow cytometric analysis. ICS analysis was performed using a FACSCalibur flow cytometer (BD) according to the methods described previously [11]. Twenty mice were tested from the group vaccinated with pVAX1-Ub-M, and five mice from the groups vaccinated with pVAX1 and PBS were tested at each time point. The splenocytes from each vaccination group were counted, pooled, and stimulated *in vitro *with M protein-derived peptides, as detailed in section *2.12*. Ten thousand CD3^+ ^T lymphocytes were acquired per sample and the number of IFN-γ-secretion CD3^+^CD8^+ ^T cells were enumerated. The CD3^+^CD8^+ ^lymphocytes that expressed IFN-γ following peptide stimulation were considered to be peptide-specific CTLs. Specific CTL responses were evaluated as the increase in the number of CD3^+^CD8^+^IFN-γ^+ ^cells. Reagents used include PerCP-conjugated CD3e, PE-conjugated CD8a and FITC-conjugated IFN-γ, and BD Cytofix/Cytoperm™, all purchased from Becton, Dickinson & Co (BD).

### 2.14. Confirmation of ICS results by ELISpot assay

To further confirm the results of the ICS, the other sixty mice from the group vaccinated with pVAX1-Ub-M and thirty mice from the groups vaccinated with pVAX1 and PBS were tested by the IFN-γ ELISPOT assay at three different time points (as detailed in section *2.13*). Data are expressed as the number of IFN-γ-secreting cells per two hundred thousand splenocytes. Peptide-specific IFN-γ ELISPOT responses were considered to be positive if the response (minus media background) was ≥ 3-fold above the media response and ≥ 50 spot-forming cells (SFC)/2 × 10^5 ^splenocytes were registered.

### 2.15. Statistical analysis

The iELISA results, the percentage of IFN-γ positive CD3^+^CD8^+ ^T lymphocytes and the number of spots per 2 × 10^5 ^splenocytes were analyzed using the analysis of variance (ANOVA), and a probability value below 0.05 was considered significant.

## 3. Results and Discussion

### 3.1. The truncated expression of PRRSV M

As the full-length M protein is difficult to express *in vitro*, we expressed a truncated version of M that lacks 84 hydrophobic amino acids at the N-terminus. SDS-PAGE analysis showed that cells transformed with the pGEX-M expression vector produced a large amount of protein with a molecular mass of approximately 36 KDa, consistent with the expected molecular weight of the truncated M protein fused with a GST tag (data not shown). Western blot analysis using an anti-PRRSV-M antibody confirmed the expression and identity of the truncated M protein fused with a GST tag (Additional file 1, Fig. S1).

### 3.2. Expression of pVAX1-Ub-M in vitro

The Ub Proteasome Pathway (UPP) is the principal mechanism for protein catabolism in the mammalian cytosol and nucleus. In order to enhance the Ub-mediated degradation efficiency of M protein, we expressed a Ub-M fusion protein in BHK-21 cells using the eukaryotic expression plasmid pVAX1-Ub-M, in which the Ub coding sequence was fused in-frame with the PRRSV M coding sequence. Following transient transfection of BHK-21 cells with the pVAX1-Ub-M plasmid, the Ub-M fusion protein was expressed and accumulated predominantly in the cytoplasm (Additional file 2, Fig. S2).

### 3.3. Identification of the recombinant vaccinia virus

Immunization strategies that prime and boost with recombinant DNA vectors encoding antigens have been shown to elicit T-cell immunity against HIV in non-human primates [18], and more recently, in humans [19]. Therefore, we used a DNA vector encoding the PRRSV M protein to immunize mice in order to generate and characterize M protein-reactive CD8^+ ^T cells. The recombinant vaccinia virus rWR-PRRSV-M drove expression of an M protein with the expected molecular weight when transfected into BHK-21 cells (Additional file 3, Fig. S3). IFA confirmed expression of M protein following transfection of BHK-21 cells, and revealed M protein accumulation in the cytoplasm (Additional file 4, Fig. S4).

### 3.5. Detection of antibodies against M protein by ELISA

M protein-specific serum antibody increased steadily from the second to the fourth DNA vaccination with the pVAX1-Ub-M vector which drives M protein expression *in vivo *(Additional file 5, Fig. S5), indicating that DNA vaccination elicited M protein-specific immune responses as expected. A subsequent booster vaccination with the recombinant vaccinia virus, rWR-PRRSV-M, elicited a further increase in PRRSV-specific antibody (P < 0.01, Additional file 5, Fig. S5). In contrast, control mice vaccinated with pVAX1-only or PBS-only did not show significant increases in M protein-specific antibody titers after the booster vaccination with rWR-PRRSV-M (P > 0.05, Additional file 5, Fig. S5). Overall, mice vaccinated with pVAX1-Ub-M generated significantly higher M protein-specific antibody titers compared to mice vaccinated with pVAX1 or PBS (P < 0.01, Additional file 5, Fig. S5). These results indicate that rWR-PRRSV-M amplifies the protective effects of DNA vaccination and reveals the advantage of this priming-boosting strategy. DNA vaccination with pVAX1-Ub-M likely drives the differentiation of memory B cells which are subsequently activated by rWR-PRRSV-M following the booster immunization, resulting in increased M-specific antibody titers. Mice vaccinated with PBS and pVAX1 would not be expected to generate M protein-reactive memory B cells, accounting for a less pronounced increase in M protein-specific antibody titers following rWR-PRRSV-M innoculation.

### 3.6. Identification of PRRSV-M CTL epitopes by ICS and ELISPOT

**Figure 1:**
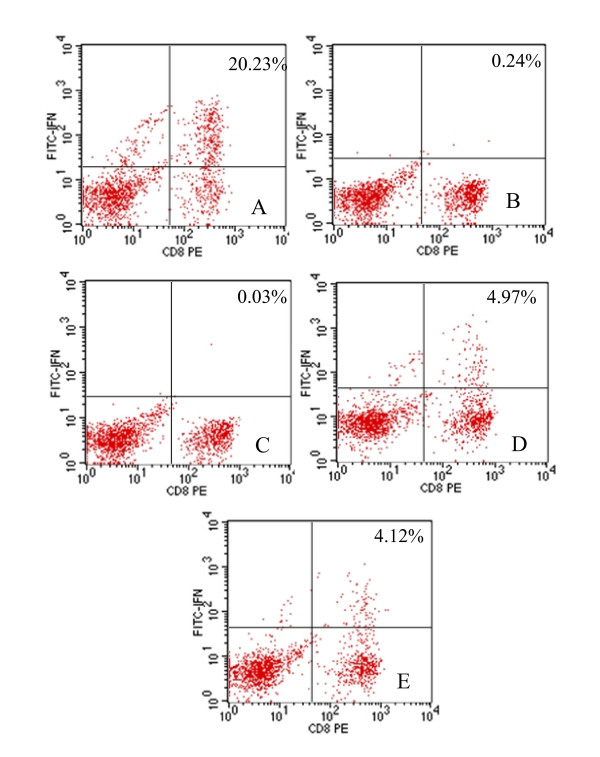
**A representative response was obtained from one mouse on 3 day after boosting with rWR-PRRSV-M, and two M protein peptides stimulate IFNγ^+ ^production in CD8^+ ^T cells of immunized BALB/c mice by ICS**. Splenocytes from immunized BALB/c mice were (A) stimulated with PMA and ionomycin: 20.23%, (B) left unstimulated: 0.24%, (C) stimulated with an irrelevant peptide: 0.03%, (D) stimulated with the peptide "K_93_**F**ITSRCR**L**": 4.97%, or (E) stimulated with the peptide "F_57_**G**YMTFVH**F**": 4.12%, and were stained with antibodies to identify IFNγ production in CD8^+ ^T cells by flow cytometry. The % in each data plot represents the percentage of CD8^+ ^T cells that secreted IFNγ.

**Figure 2:**
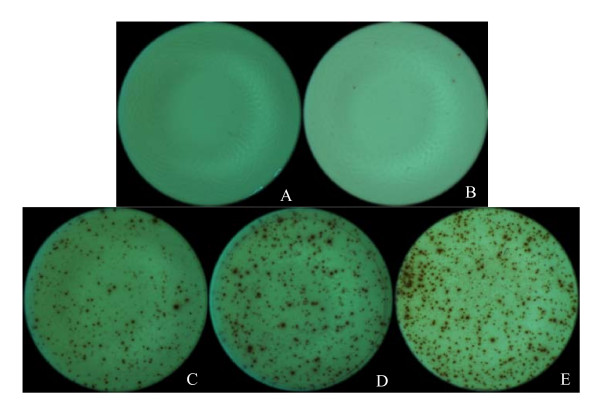
**Two M protein peptides stimulate IFNγ^+ ^production in splenocytes from immunized BALB/c mice in an ELISPOT assay**. Splenocytes from immunized BALB/c mice were (A) left unstimulated, (B) stimulated with an irrelevant peptide, (C) stimulated with the peptide "F_57_**G**YMTFVH**F**": 54 spots/5 × 105 cells, (D) stimulated with the peptide "K_93_**F**ITSRCR**L**": 81 spots/5 × 105 cells, or (E) stimulated with PMA and ionomycin (324 spots/5 × 105 cells) to determine the frequency of IFNγ-secreting splenocytes.

In this study we identified potential CTL epitopes in BALB/c mice vaccinated with pVAX1-Ub-M and boosted with rWR-PRRSV-M. The frequency and number of CD3^+^CD8^+ ^T cells that produced IFN-γ following stimulation with peptides derived from PRSSV M protein was enumerated using ICS and ELISPOT assays. Using these approaches, we identified two peptides from PRSSV M protein that elicited IFN-γ production from the splenocytes of vaccinated mice. As shown in Figure 1, intracellular IFN-γ staining following stimulation of splenocytes from vaccinated mice with the peptide K_93_FITSRCRL and F_57_GYMTFVHF revealed a population of IFN-γ producing CD8^+ ^T cells comprising 4-5% of the total CD3^+ ^splenocyte population. In contrast, unstimulated splenocytes and splenocytes exposed to an irrevelant peptide did not contain a population of IFN-γ producing CD8^+ ^T cells (Figure 1, panel B and C). Consistent with the ICS data, peptides K_93_FITSRCRL and F_57_GYMTFVHF elicited IFN-γ production from splenocytes of vaccinated mice when measured by ELISPOT, whereas unstimulated splenocytes and splenocytes stimulated with an irrelevant peptide did not reveal IFN-γ producing cells (Figure 2). The K_93_FITSRCRL and F_57_GYMTFVHF PRSSV M protein peptides were identified bioinformatically as H-2K*^d ^*and H-2D*^d ^*restricted CTL epitopes (Table 2).

**Figure 3:**
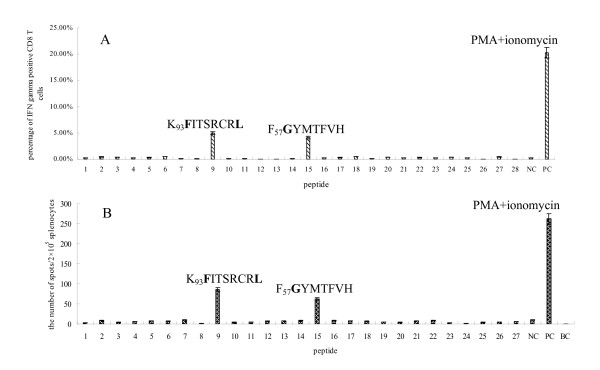
**A: Summary results of ICS analysis for IFNγ^+ ^production in CD8^+ ^T cells of immunized BALB/c mice stimulated with a panel of M protein-derived peptides**. B: Summary results of ELISPOT analysis for IFNγ^+ ^production from splenocytes of immunized BALB/c mice stimulated with a panel of M protein-derived peptides. Finally, one hundred and twenty mice were used to analyze the results from ICS and ELISPOT. And the same pattern of reactivity on days 3, 5 and 12 after boosting with rWR-PRRSV-M within each vaccination group were analyzed, and the numbers 1-27 represent different peptides derived from M protein of PRRSV Ch-1a strain; Number 28 is an irrelevant peptide control; NC is an unstimulated negative control; PC is a PMA + ionomycin stimulated positive control; BC is a background control. Data in the bar graphs (A and B) are means and standard deviations from the group vaccinated with pVAX1-Ub-M.

Specific increases in the number of cells producing IFN-γ following stimulation with the peptides "K_93_FITSRCRL" and "F_57_GYMTFVHF" was observed by day 3 after the booster vaccination with rWR-PRRSV-M (Figure 1 and 2). Furthermore, splenocytes from mice vaccinated with pVAX1-Ub-M responded strongly to "K_93_FITSRCRL" and "F_57_GYMTFVHF", but not other M protein-derived peptides, at all time points tested following the booster vaccination with rWR-PRRSV-M. When the same pattern of reactivity on three different time points after boosting with rWR-PRRSV-M within each vaccination group were analyzed statistically using ANOVA analysis, statistically significant differences were noted for the peptides "K_93_FITSRCRL" and "F_57_GYMTFVHF" when compared to the other peptides tested among mice vaccinated and boosted with pVAX1-Ub-M and rWR-PRRSV-M, respectively (P < 0.01, Figure 3A and 3B). Conversely, and confirming the specificity of these responses, no difference among the stimuli was observed with mock-vaccinated (pVAX1) mice and naïve mice (data not shown).

It is important to recognize that the ICS assay calculates the percentage of IFN-γ^+ ^cells among CD3^+ ^T cells in the spleen (4-5%), whereas the ELISPOT assay assesses the number of IFN-γ^+ ^cells among all splenocyte cell types (0.05%), and cannot definitively assign the production of IFN-γ to a particular cell type. Thus, the two assays use different denominators in calculating the frequency of IFN-γ^+ ^production by splenocytes. Importantly, each method clearly identified K_93_FITSRCRL and F_57_GYMTFVHF as the only two peptides from a panel of 27 that elited significant IFN-γ production from the splenocytes of vaccinated mice.

In this study we used BALB/c mice as a model system to identify CTL epitopes in the M protein of PRSSV to circumvent limitations derived from shortages of inbred pigs and a paucity of reagents to evaluate porcine immune responses. The identification of PRSSV CTL epitopes in BALB/c mice allow for the future generation of reagents, such as MHC class I: peptide staining reagents, that will enable the in-depth investigations of CD8^+ ^T cell responses during PRSSV infection and immuinization. Whether these two epitopes can bind the SLA class I molecules of pigs remains to be determined. In some cases, specific peptide epitopes are known to be recognized by cytotoxic T cells in different animal species. For instance, the core region of HCV contains an epitope that is recognized by cytotoxic T cells of both mice and humans [19]. Further research on the role of these peptide epitopes in different species is ongoing in our laboratory.

In conclusion, we identified peptides "K_93_FITSRCRL" and "F_57_GYMTFVHF" from the M protein of PRSSV as H-2K*^d ^*and H-2D*^d ^*restricted CTL epitopes, respectively. In this study, we also developed a mouse model of PRRSV infection and this will undoubtedly contribute to our understanding of the cell-mediated immune responses to PRRSV.

## Competing interests

The authors declare that they have no competing interests.

## Authors' contributions

DLW and GLL gave me the idea of this study. WJZ and YL participated in the design and conducted the majority of the experiments as well as drafted the manuscript. TGT helped revise the manuscript and participated in the first stage of the experiments. YB and QW participated in the prediction of CTL epitopes and analyzed the data. YHX, NHL and TY participated in the sequence alignment. ZGB provided the expression system of recombinant vaccinia virus, and GZT provided the PRRSV Ch-1a strain. All the authors read and approved the final manuscript.

## Supplementary Material

Additional file 1 **Western blotting results of the recombinant protein PRRSV-M expressed in E.coli**. **Fig.S1. **Western blotting analysis of the recombinant protein PRRSV-M expressed in *E.coli *BL21 (DE3) cells transformed with the pGEX-M expression vector. SDS-PAGE analysis showed that cells transformed with the pGEX-M expression vector produced a large amount of protein with a molecular mass of approximately 36 KDa, consistent with the expected molecular weight of the truncated M protein fused with a GST tag (data not shown). And western blot analysis using an anti-PRRSV-M antibody confirmed the expression and identity of the truncated M protein fused with a GST tag (Lane 1), while there was no such signal at the corresponding position of the negative control sample (Lane 2). Lane M. prestained protein mass marker; Lane 1. recombinant PRRSV M protein; Lane 2. pGEX-6P-1. Click here for file

Additional file 2 **IFA results of eukaryotic expression plasmid pVAX1-Ub-M in transfected BHK-21 cells**. **Fig.S2. **IFA result of BHK-21 cells transfected with pVAX1-U-M. BHK-21 cells were transfected with pVAX1-U-M or empty pVAX1 plasmids. After 36 hours, IFA was performed using an M protein-specific antibody. A. pVAX1-U-M; B. pVAX1. (magnifications are × 200). Click here for file

Additional file 3 **Western blot results of recombinant vaccinia virus rWR-PRRSV-M in infected BHK-21 cell**. **Fig.S3. **Western blot analysis of BHK-21 cell lysates following infection with rWR-PRRSV-M. BHK-21 cells were infected with rWR-PRRSV-M or WR strain vaccinia virus. After 72 hours, cell lysates were generated for Western blot using an M protein-specific antibody. The results showed that the recombinant vaccinia virus rWR-PRRSV-M drove expression of a complete M protein with the expected molecular weight (17 KDa) when transfected into BHK-21 cells. Lane M: prestained protein mass marker; Lane 1. Lysate from cells infected with rWR-PRRSV-M; Lane 2. Lysate from cells infected with WR strain vaccinia virus. Click here for file

Additional file 4 **IFA results of recombinant vaccinia virus rWR-PRRSV-M and vaccinia virus WR strain in infected BHK-21 cell**. **Fig.S4. **IFA result of BHK-21 cells infected with rWR-PRRSV-M and vaccinia virus WR strain. BHK-21 cells were infected with (A) rWR-PRRSV-M or (B) vaccinia virus WR strain. After 72 hours, IFA was performed using an M protein-specific antibody. (magnifications are × 100). Click here for file

Additional file 5 **ELISA antibody response in mice after immunization following DNA vaccination and a booster vaccination with recombinant vaccinia virus**. **Fig.S5. **M protein-specific antibody responses in mice immunized with PBS, or pVAX1 or pVAX1-U-M DNA, and boosted with rWR-PRRSV-M. Serum samples was obtained from vaccinated mice 7 days after each DNA vaccination and 3 days after boosting with rWR-PRRSV-M, and were evaluated for reactivity to M-protein in an ELISA based on coating with the truncated M protein fused with a GST tag. And, the day 0 represents the day of the first DNA immunization. Statistically significant differences are indicated by "*" or "**" for p-values < 0.05 or < 0.01, respectively, as determined by ANOVA. Click here for file
