# Supplementary figures and images for: Identification of CD8+ cytotoxic T lymphocyte epitopes from porcine reproductive and respiratory syndrome virus matrix protein in BALB/c mice
Source: Virol J. 2011 May 30;8:263. doi: 10.1186/1743-422X-8-263 (PMC3126774; doi:10.1186/1743-422X-8-263)

70kDa

55kDa

40kDa

35kDa

25kDa

15kDa

36kDa

2

1

M


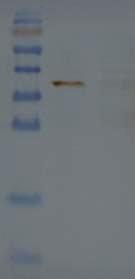


10kDa

Supplement: Additional file 1 — Western blotting results of the recombinant protein PRRSV-M expressed in E.coli. Fig.S1. Western blotting analysis of the recombinant protein PRRSV-M expressed in E.coli BL21 (DE3) cells transformed with the pGEX-M expression vector. SDS-PAGE analysis showed that cells transformed with the pGEX-M expression vector produced a large amount of protein with a molecular mass of approximately 36 KDa, consistent with the expected molecular weight of the truncated M protein fused with a GST tag (data not shown). And western blot analysis using an anti-PRRSV-M antibody confirmed the expression and identity of the truncated M protein fused with a GST tag (Lane 1), while there was no such signal at the corresponding position of the negative control sample (Lane 2). Lane M. prestained protein mass marker; Lane 1. recombinant PRRSV M protein; Lane 2. pGEX-6P-1. [file 1743-422X-8-263-S1.DOC]

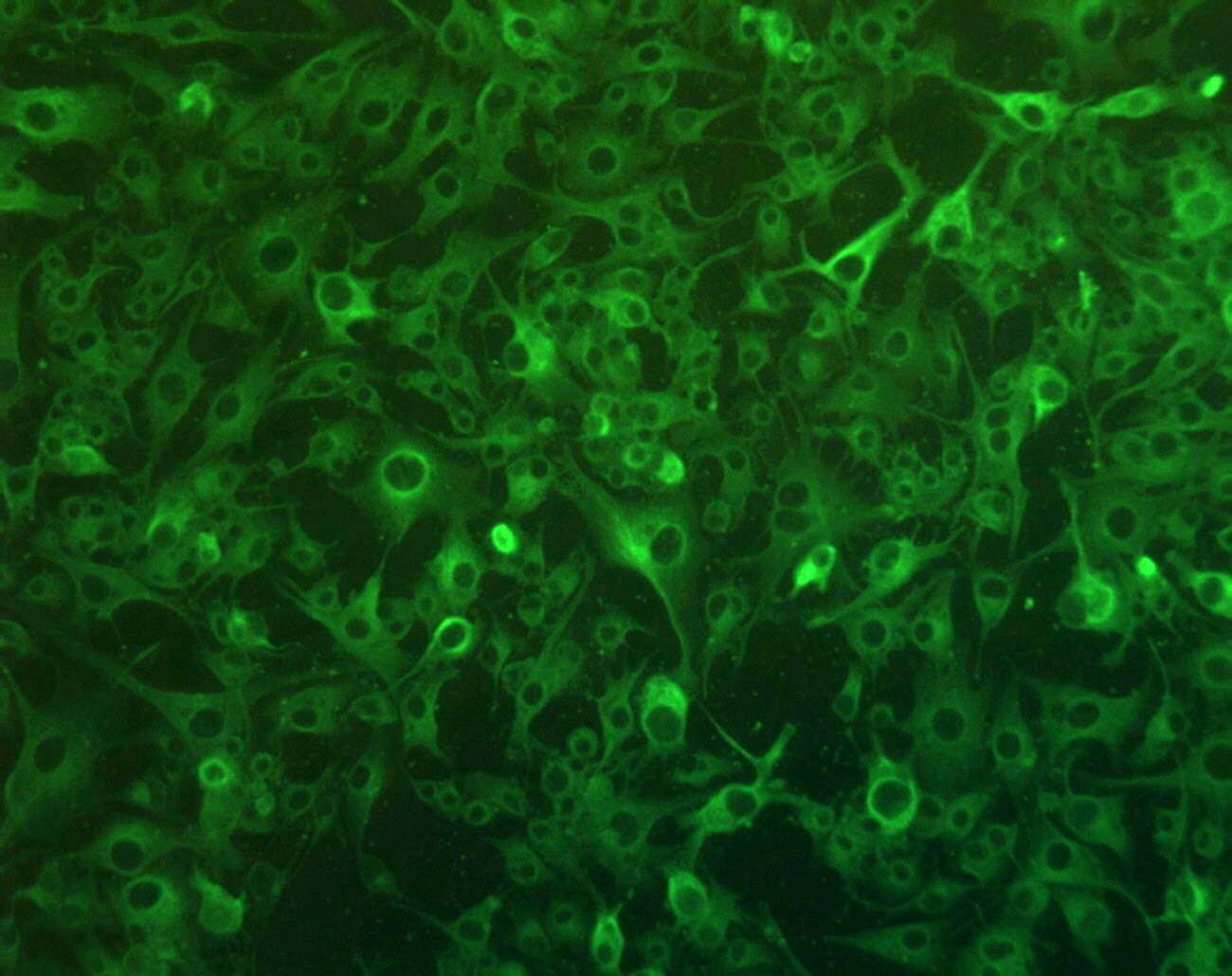


A


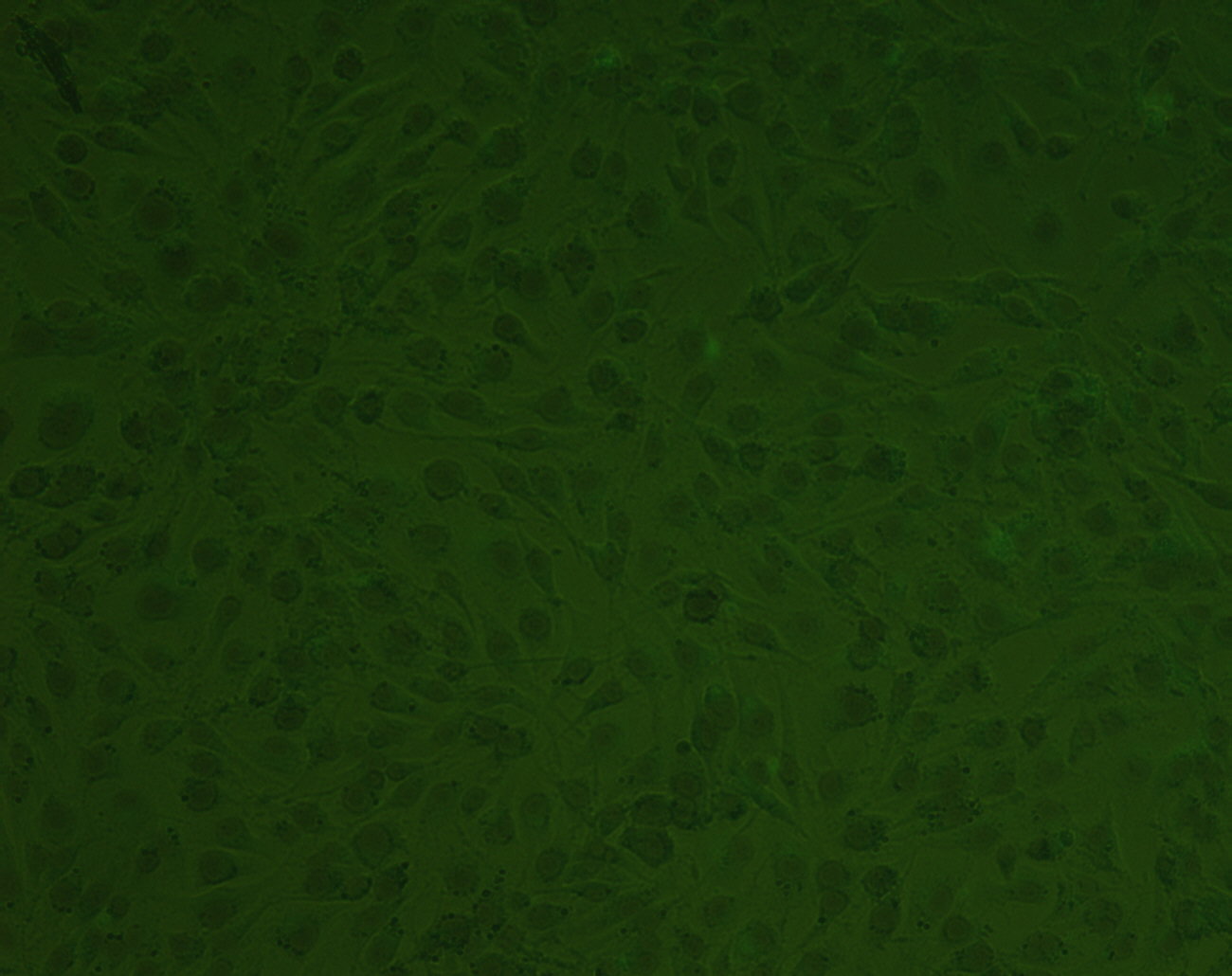


B

Fig.2: IFA result of BHK-21 cells transfected by pVAX1-M and pVAX1-U-M.

A: pVAX1-U-M; C: pVAX1.

Supplement: Additional file 2 — IFA results of eukaryotic expression plasmid pVAX1-Ub-M in transfected BHK-21 cells. Fig.S2. IFA result of BHK-21 cells transfected with pVAX1-U-M. BHK-21 cells were transfected with pVAX1-U-M or empty pVAX1 plasmids. After 36 hours, IFA was performed using an M protein-specific antibody. A. pVAX1-U-M; B. pVAX1. (magnifications are × 200). [file 1743-422X-8-263-S2.DOC]

19kDa

2

1

M

100kDa

70kDa

55kDa

40kDa

35kDa

25kDa

15kDa


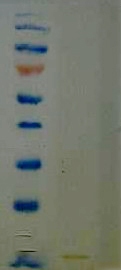

Supplement: Additional file 3 — Western blot results of recombinant vaccinia virus rWR-PRRSV-M in infected BHK-21 cell. Fig.S3. Western blot analysis of BHK-21 cell lysates following infection with rWR-PRRSV-M. BHK-21 cells were infected with rWR-PRRSV-M or WR strain vaccinia virus. After 72 hours, cell lysates were generated for Western blot using an M protein-specific antibody. The results showed that the recombinant vaccinia virus rWR-PRRSV-M drove expression of a complete M protein with the expected molecular weight (17 KDa) when transfected into BHK-21 cells. Lane M: prestained protein mass marker; Lane 1. Lysate from cells infected with rWR-PRRSV-M; Lane 2. Lysate from cells infected with WR strain vaccinia virus. [file 1743-422X-8-263-S3.DOC]

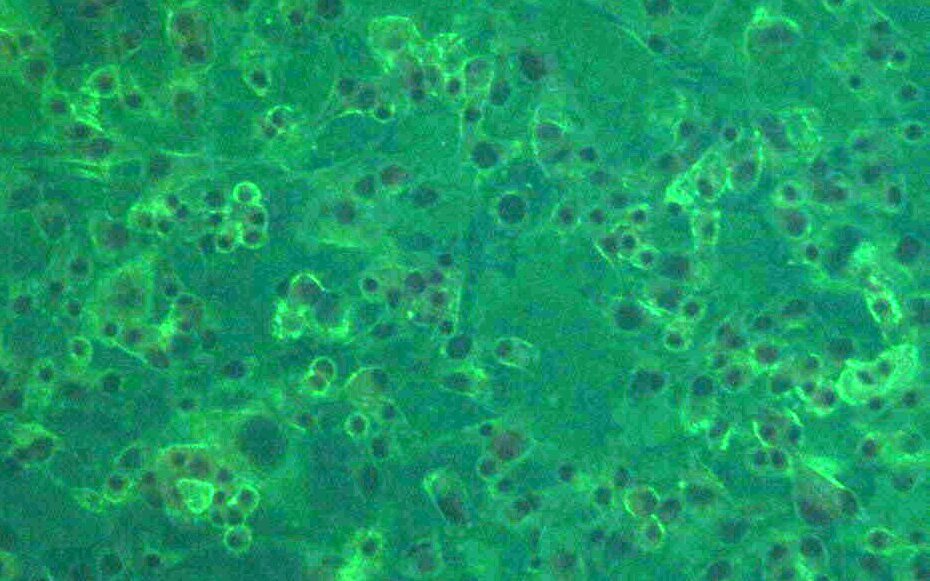


A


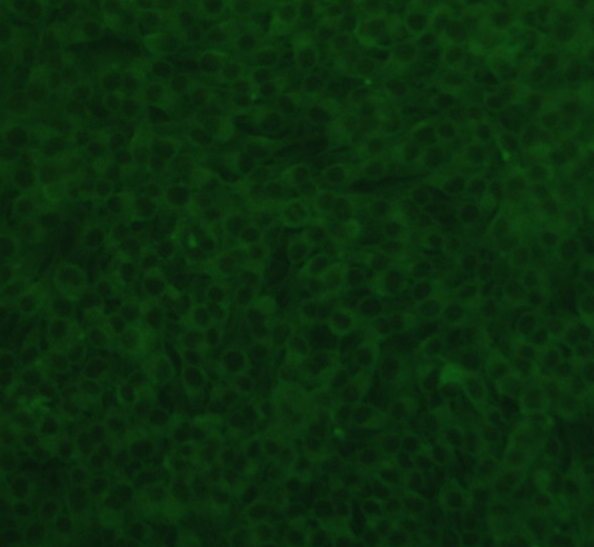


B

Supplement: Additional file 4 — IFA results of recombinant vaccinia virus rWR-PRRSV-M and vaccinia virus WR strain in infected BHK-21 cell. Fig.S4. IFA result of BHK-21 cells infected with rWR-PRRSV-M and vaccinia virus WR strain. BHK-21 cells were infected with (A) rWR-PRRSV-M or (B) vaccinia virus WR strain. After 72 hours, IFA was performed using an M protein-specific antibody. (magnifications are × 100). [file 1743-422X-8-263-S4.DOC]
