# Supplementary material for: Identification of CD8+ cytotoxic T lymphocyte epitopes from porcine reproductive and respiratory syndrome virus matrix protein in BALB/c mice
Source: Virol J. 2011 May 30;8:263. doi: 10.1186/1743-422X-8-263 (PMC3126774; doi:10.1186/1743-422X-8-263)
Supplement: Additional file 5 — ELISA antibody response in mice after immunization following DNA vaccination and a booster vaccination with recombinant vaccinia virus. Fig.S5. M protein-specific antibody responses in mice immunized with PBS, or pVAX1 or pVAX1-U-M DNA, and boosted with rWR-PRRSV-M. Serum samples was obtained from vaccinated mice 7 days after each DNA vaccination and 3 days after boosting with rWR-PRRSV-M, and were evaluated for reactivity to M-protein in an ELISA based on coating with the truncated M protein fused with a GST tag. And, the day 0 represents the day of the first DNA immunization. Statistically significant differences are indicated by "*" or "**" for p-values < 0.05 or < 0.01, respectively, as determined by ANOVA. [file 1743-422X-8-263-S5.DOC]

*

*

*

**
